# Reconstruction of a Lower Polar Artery for Kidney Transplantation Using Donor Ovarian Vein: Case Report with Literature Review

**DOI:** 10.3390/medicina57111248

**Published:** 2021-11-15

**Authors:** Saulė Bikauskaitė, Kamilė Počepavičiūtė, Linas Velička, Antanas Jankauskas, Darius Trumbeckas, Erika Šuopytė

**Affiliations:** 1 Faculty of Medicine, Medical Academy, Lithuanian University of Health Sciences, LT-44307 Kaunas, Lithuania; 2 Department of Radiology, Medical Academy, Lithuanian University of Health Sciences, LT-44307 Kaunas, Lithuania; kamile.pocepaviciute@stud.lsmu.lt; 3 Clinic of Vascular Surgery, Hospital of Lithuanian University of Health Sciences Kauno Klinikos, LT-50161 Kaunas, Lithuania; linas.velicka@lsmu.lt; 4 Department of Radiology, Hospital of Lithuanian University of Health Sciences Kauno Klinikos, LT-50161 Kaunas, Lithuania; antanas.jankauskas@lsmuni.lt; 5 Department of Urology, Hospital of Lithuanian University of Health Sciences Kauno Klinikos, LT-50161 Kaunas, Lithuania; darius.trumbeckas@lsmu.lt (D.T.); erika.suopyte@gmail.com (E.Š.)

**Keywords:** kidney transplantation, accessory renal artery injury, arterial reconstruction, ovarian vein, polar artery

## Abstract

*Background:* In the case of complicated kidney transplantation, when the accessory artery is severed, the main task is to decide whether to restore renal blood flow and which method should be used. In this report, we present a case of kidney transplantation with vascular reconstruction using an ovarian vein as an interposition graft between a larger branch of the main renal artery and the lower polar artery which was severed during kidney explantation. *Case summary:* Kidney transplantation using an ovarian vein was performed for a 34-year-old woman with end-stage renal disease on 1 April 2020 in the Hospital of Lithuanian university of health sciences (LUHS) Kaunas Clinics. A lower accessory renal artery was severed during kidney explantation. As the ovarian vein of the donor remained and matched the diameter of the severed vessel, it was decided to use it as an insertion between the main renal artery and the accessory renal artery of the inferior pole. The cold ischemic time was 770 min and the warm ischemic time was 37 min. A month after transplantation, the patient’s condition and daily urine output were normal and the serum creatinine level decreased rapidly. Fifteen months after the surgery, the function and structure of the transplant remained normal and there was no evidence of serious vascular complications on CT scans. This is the first case where graft function was verified after transplantation using three-dimensional CT angiography. *Conclusions:* If an inferior polar artery is severed, vascular reconstruction must be performed to preserve the function of the graft. Usually, the gonadal vein is available during donor nephrectomy; therefore, it can be explanted without additional difficulties or incisions. Although we have not reported any complications, further studies are recommended on the long-term outcomes of this alternative approach for the reconstruction of short renal arteries.

## 1. Introduction

Kidney transplantation is one of the most effective treatments for end—stage renal disease. Compared to dialysis, successful transplantation is associated with better quality of life and lower mortality risk [1]. However, to minimize warm and cold ischemia, the urgency of the operation is increased. Therefore, during renal explantation, the possibility of transplant injury occurs. According to the study by Copelan et al. on the occurrence of iatrogenic-related transplant injuries, 7.1% of explantations are related to vascular (4.8%), capsular (1.7%), and ureteric (0.7%) injuries [2]. The risk of vascular injury increases with the presence of a polar artery, which is observed in 12–25% of the general population [3]. The accessory renal artery is a vestigial formation that persists due to its malfunction in degeneration during the ascent of the metanephros [4]. The renal arteries develop from a single middle pair of lateral mesonephric arteries which arise from the dorsal aorta. Accessory renal arteries may originate from the remaining arteries of the middle group [5]. Based on the origin of branching, the classification of polar arteries was made in 1958 by Merklin and Michels. In most cases, polar arteries arise from the abdominal aorta; however, accessory vessels of the kidney may also originate from the main renal artery or the external iliac, inferior phrenic, superior suprarenal, right colic, inferior mesenteric, lumbar, gonadal, subcostal, splenic arteries, and thoracic aorta [6]. Accessory renal arteries may be unilateral or bilateral and occur in different numbers (usually one or two) [7]. Nevertheless, there are some possibilities of vascular reconstruction for harvesting injuries. Direct anastomosis with other arteries or the vascular interposition graft method can be used to restore renal blood flow. Smaller polar arteries result in minor infarcts which are not harmful enough to require vascular repair. This is usually the case with the upper polar artery. However, due to the blood supply to the ureter of the graft, lower polar arterial injuries are more serious and clinically significant [8]. In the case of a complicated kidney transplantation, when the accessory artery is severed, the main task is to decide whether to restore renal blood flow and which method should be used. This article aims to present a case of kidney transplantation with vascular reconstruction using an ovarian vein as an interposition graft between a larger branch of the main renal artery and the lower polar artery which was severed during kidney explantation.

## 2. Case Report

Here, we describe a successful case of kidney transplantation using an ovarian vein, performed for a 34-year-old woman with end-stage renal disease on 1 April 2020 in the Hospital of LUHS Kaunas Clinics. The recipient was diagnosed with Alport syndrome and membranoproliferative glomerulonephritis as well. A kidney was available from a 53-year-old deceased female donor who died from a hemorrhagic stroke. The function of the donor’s kidney was normal.

Pre-transplant evaluation of the donor’s kidney function showed a normal level of serum creatinine, urea, and potassium. The results of renal ultrasonography showed normal size and structure of the kidney, normal renal parenchymal echogenicity and thickness, and no signs of dilation in the urinary collecting system.

This case presented several fundamental problems. Initially, an accessory renal artery was severed during explantation. This vessel was large (about 3 mm in diameter) and fed a part of the inferior kidney pole. Furthermore, all four renal arteries arose from a very short (about 5 mm in length) common trunk. As the ovarian vein of the donor remained and matched the diameter of the severed accessory artery, it was decided to use it as an insertion between the main renal artery and the accessory renal artery of the inferior pole.

**Figure 1:**
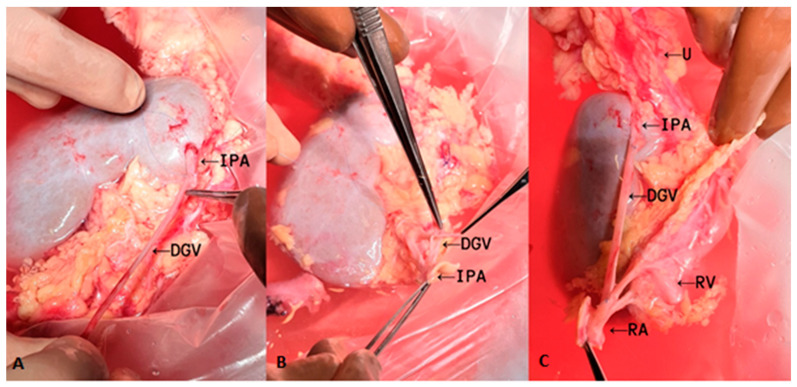
Reconstruction of the inferior polar artery with the ovarian vein on the back table. (**A**) Distal end-to-end anastomosis of an interposition graft; (**B,C**) proximal end-to-side anastomosis of an interposition graft. IPA, inferior polar artery; DGV, donor gonadal vein; U, ureter; RV, renal vein; RA, renal artery.

The inferior polar artery was reconstructed on the back table (Figure 1). End-to-end anastomosis with the reversed ovarian vein and a short segment of the polar artery was performed with 7—0 Prolene running suture. Proximal anastomosis of the inverted ovarian vein and one of the four renal arteries from the common trunk was performed in an end-to-side manner. The transplant surgery was performed in the left iliac fossa. Firstly, the renal vein was anastomosed to the external iliac vein. Then, an end-to-side anastomosis was made between the common trunk of the renal artery and the external iliac artery. Subsequently, the kidney was reperfused. The cold ischemic time (from kidney perfusion with cold solution after the nephrectomy to physiological temperature of the transplant during implantation procedures) was 770 min and the warm ischemic time (from physiological temperature before the completion of the vascular anastomosis to the reestablishment of the kidney blood circulation) was 37 min.

A month after the transplantation patient’s condition and daily urine output were normal, the serum creatinine level decreased rapidly. The levels of serum creatinine and leukocytes in urine were significantly reduced (Table 1). The results of renal ultrasonography showed normal size and structure of the kidney, with a parenchymal thickness of 1.6 cm. Renal transplant blood flow was without abnormalities and segmental defects. The renal arterial resistive index was 0.7. The urinary collecting system was without signs of dilation.

**Figure 2:**
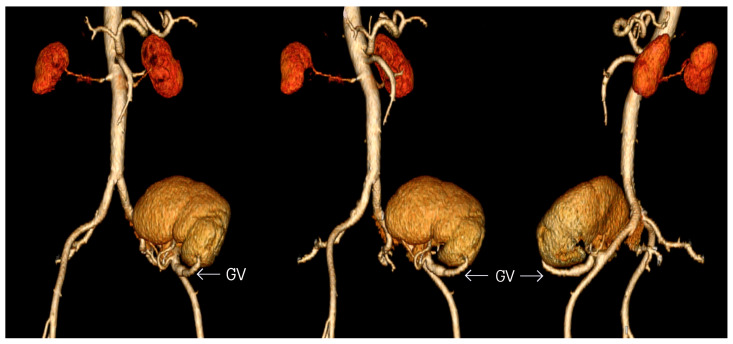
CT angiography with aortoiliac reconstruction after kidney transplantation. GV, gonadal vein.

The kidney transplant was reassessed 15 months after the surgery. Its function and structure remained normal. Although CT scans showed thickening of transplanted kidney’s arterial walls, there was no other evidence of vascular complications. Additionally, there were no defects of contrast enhancement in the parenchyma. This is the first case where graft function was verified after transplantation using three-dimensional CT angiography (Figure 2). The results of dynamic renal scintigraphy and renal biopsy were without abnormalities too.

The Medical Subject Headings (MeSH) which were selected to represent this case report are specified in Table A1 (Appendix A).

## 3. Discussion

Kidney transplantation with a polar artery can be challenging due to the possibility of errors during explantation. Novick et al. reported that unilateral (23%) polar arteries are unrecognized and injured more often than bilateral ones (10%). This pattern can be explained by increased attention when a first polar artery is observed [9].

The necessity of reconstruction of an accessory renal artery depends on the supplying area of the kidney. If it is less than a quarter, it will not affect the function of the graft. However, this rule can be applied only for an upper polar artery because a lower polar artery is significant for the ureter blood supply and necessary for the healing after a ureterocystoneostomy. Successful attachment of the ureter and adequate blood flow prevent urological complications such as urinary leakage [8]. In our case, the lower accessory artery, which fed a significant part of the inferior kidney pole, was damaged; therefore, it was decided to perform vascular reconstruction. Without this procedure, the graft would no longer be usable due to ischemic complications, which can lead to necrosis and kidney failure [10].

However, the possibility of complications remains when performing vascular reconstruction. The results of a meta-analysis published by Afriansyah et al. showed that stenosis and thrombosis of vessels occur more often in the presence of a polar artery compared with a single main kidney artery [11]. Reconstruction of a polar artery using back-table preparation prolongs the cold ischemia time, which can lead to delayed graft function (DGF). The risk increases if the cold ischemia lasts longer than 12 h. According to some studies, delayed graft function (DGF) is associated with an increased risk of acute rejection, extended time of hospitalization, and the possibility of long-term outcomes after kidney transplantation. Since the duration of cold ischemia in a graft with an accessory artery is approximately 80 min and in a single artery allograft is 70 min, statistical significance has not been established [11,12]. If back-table preparation is not used, allograft transplantation with separate vascular anastomosis of the polar artery to the external iliac artery can prolong the warm ischemia time, resulting in an increased risk of acute tubular necrosis and decreased graft function [13]. Within 20 min of removing the kidney from the ice, the temperature can quickly increase above the metabolic threshold of 15 °C [14]. According to Weissenbacher et al., warm ischemia lasting more than 30 min needs to be considered as a major risk factor for the long-term results [15]. Therefore, in our case, back-table vascular reconstruction was performed. To minimize the warm ischemia time, the distal end of the interposition graft was anastomosed to one of the four renal arteries from a common trunk.

Another question concerns which method of vascular reconstruction should be used. It depends on several variables: the compatibility of the diameters of the vessels, the length of polar artery segment, and the presence of atherosclerosis or calcification of vessels [16]. There are several methods of vascular reconstruction when an accessory artery is damaged. If the distal end of the polar artery is preserved, direct repair by connecting two interrupted segments of the artery can be used. However, this method is possible only if the aortic patch with polar artery bifurcation is retained. If the distal end of the polar artery is injured, it can be reimplanted into the trunk of the main renal artery or a larger vessel such as an external iliac artery, internal iliac artery, or hypogastric artery. Another method is to create a common ostium by making a side-to-side conjoined anastomosis with another renal artery. However, such cases with direct polar artery reimplantation are very rare and the artery length is usually insufficient; therefore, vascular interposition grafts are used [17]. According to a study which compared all methods of vascular reconstruction, the warm and total ischemia times of reconstruction with interposition graft were significantly longer than in the nonarterial reconstruction group. However, the time to initial urination, perioperative and postoperative estimated glomerular filtration rate (eGFR), and complications were similar regardless of the method [18].

A vascular interposition graft can be made from a donor or a recipient’s vessels. Even though the saphenous vein is usually associated with coronary or lower extremity vascular bypass graft, it can be used as an interposition graft in renal vessels as well. Oertl et al. reported that no additional intraoperative complications were observed, and intraoperative blood loss was not significantly elevated in the group with saphenous vein interposition compared to kidney transplantation without vascular reconstruction [19]. However, due to thrombosis, intimal hyperplasia, and atherosclerotic degeneration, saphenous vein graft has a higher risk of occlusion and long-term complications such as aneurysms and ruptures. Furthermore, access to the vein requires an additional incision [20,21]. Internal iliac artery graft is usually used in cases with three or more polar arteries and is taken from the recipient. It is reported as a safe and easy technique as each graft artery is anastomosed with each branch of the recipient’s iliac artery during back-table preparation and reanastomosed to the recipient’s primary iliac artery with the same diameter [18]. Another option is using the inferior epigastric artery, which can be anastomosed after the restoration of the graft circulation. This method is superior for decreasing the ischemia time and has no donor-site complications [22]. No reports on arteriosclerosis or other long-term complications have been reported. Polytetrafluoroethylene (PTFE) vascular graft can be used in rare cases too when other biologic vascular graft options are not available. However, there are not enough studies about PTFE long-term complications in reconstructing damaged renal vessels during kidney transplantation, and most of the long-term data are about PTFE vascular grafts in lower limb revascularization [23]. Nevertheless, prosthetic vascular grafts have the highest rate of thrombogenicity and infectability compared to biologic vascular interposition grafts [24]. The advantages and disadvantages of different techniques of vascular reconstruction when the polar artery is severed are represented in Table 2.

In our case, the ovarian vein was used due to the preservation during explantation and the matching diameter of the severed accessory artery. For a period of 11 years, we found just four other reported cases in which use of the gonadal vein for interposition graft was mentioned (Table 3) [6,25,26,27]. None of those had any short- or medium-term complications. The research shows that there are no differences in urological complications between preserved or dissected gonadal veins from recipients [28]. Although long-term complications have not been reported yet, patients should be monitored further for graft stenosis and aneurysm.

## 4. Conclusions

If the inferior polar artery is severed, vascular reconstruction must be performed to preserve the function of the graft. Usually, the gonadal vein is available during donor nephrectomy; therefore, it can be explanted without additional difficulties or incisions. In this case, the ovarian vein was used as an interposition graft between a short segment of the severed accessory artery and the common trunk of the renal artery. To minimize the warm ischemia time, both anastomoses were made on the back table. Although we have not reported any complications, further studies are recommended on the long-term outcomes of this alternative approach for the reconstruction of short renal arteries.

## Figures and Tables

**Table 1:** Comparison of laboratory results before and after transplantation.

	Before Transplantation	1 Month after Transplantation	15 Months after Transplantation
Creatinine (µmol/L)	9710 (↑↑↑)	1750 (↑↑)	95 (↑)
Urea (mmol/L)	17.3 (↑)	7.9	7.1
Potassium (mmol/L)	5.43 (↑)	4.53	4.06
Urine pH	8.0	7.5	6.5
Urine-specific gravity	1012	1011	1006
Blood in urine	-	-	0.3
Leukocytes in urine	500 (↑↑↑)	25 (↑)	-
Erythrocytes in urine (microscopic urinalysis)	-	-	-
Leukocytes in urine (microscopic urinalysis)	273 (↑↑↑)	56 (↑)	-

↑↑↑—severely increased; ↑↑—moderately increased; ↑—mildly increased.

**Table 2:** Different techniques of vascular reconstruction.

Vascular Reconstruction Techniques of Severed Polar Artery	Indication	Method	Advantages	Disadvantages
Ligation	Upper polar artery which supplies < 25% is severed	Ligation	Does not affect the function of the graft	Cannot be applied for the lower polar artery
Direct repair	Distal end of the polar artery is preserved	Connection of two interrupted segments of the polar artery	Does not require additional vessels	Possible only if the aortic patch with polar artery bifurcation is retained
End-to-side anastomosis	Distal end of the polar artery is severed	End-to-side anastomosis with the main renal artery, external iliac artery, or internal iliac artery	The diameters of vessels can be different	If back-table preparation is not used, warm ischemia time is prolonged Higher risk of stenosis and thrombosis
End-to-end anastomosis	Distal end of the polar artery is severed	End-to-end anastomosis with hypogastric artery	Can be anastomosed after the main renal artery is reperfused Reduces ischemia time	The hypogastric artery is not always available due to atherosclerosis The size or diameter can be insufficient
Side-to-side conjoined anastomosis	Two equal-sized arteries	Common ostium is made with another renal artery	Reduction in warm ischemia time due to single artery anastomosis	Arteries need to be comparable in size
Vascular interposition graft	Insufficient artery length	Vascular interposition graft is made from donor or recipient’s vessel (saphenous vein, internal iliac artery, inferior epigastric artery, or gonadal vein) or PTFE; it is anastomosed to the severed polar artery and a larger vessel	No additional intraoperative or donor-site complications Decreases warm ischemia time due to back—table preparation	Saphenous vein graft has a higher risk of occlusion, aneurysms, and ruptures, and access to the vein requires an additional incision PTFE has the highest rate of thrombogenicity and infectability

**Table 3:** Summary of previous reports of arterial reconstruction using a gonadal vein.

Authors	Year	Age	Sex	Diagnosis	Donor’s Kidney	Number of Allograft Renal Arteries	Source of Gonadal Vein	Interposition GraftAnastomosed to	Follow-Up Period
Chatzizacharias NA	2010	28	M	IgA nephropathy	Left	2	Donor	External iliac artery	NA
Veeramani M	2010	49	M	ADPKD, ESRD	Left	2	Donor	External iliac artery	2 years
Uysal E	2017	27	M	ESRD	Right	1	Recipient	Internal iliac artery	8 months
Tomizawa M	2020	34	M	ESRD	Right	3	Donor	Graft made from the internal iliac artery	3 years
Present case	2020	34	F	Alport syndrome, MPGN, ESRD	Right	2	Donor	One of four renal arteries from a common trunk	15 months

ADPKD—autosomal dominant polycystic kidney disease; ESRD—end-stage renal disease; F—female; M—male; MPGN—membranoproliferative glomerulonephritis; NA—not available.

## Data Availability

The data presented in this study are available on request from the corresponding author. The data are not publicly available due to privacy.

## Abbreviations

ADPKD	Autosomal dominant polycystic kidney disease
CT	Computed tomography
DGF	Delayed graft function
DGV	Donor gonadal vein
eGFR	Glomerular filtration rate
ESRD	End-stage renal disease
GV	Gonadal vein
IPA	Inferior polar artery
LUHS	Lithuanian university of health sciences
MPGN	Membranoproliferative glomerulonephritis
NA	Not available
PTFE	Polytetrafluoroethylene
RA	Renal artery
RV	Renal vein
U	Ureter

## Appendix A

**Table A1:** MeSH terms.

Adult
Anastomosis, surgical/methods*
Case reports
Cold ischemia
Computed tomography Angiography
Female
Follow-up studies
Gonads/blood supply
Graft survival
Humans
Kidney failure, chronic/surgery
Kidney function Tests
Kidney transplantation/methods*
Lithuania
Nephrectomy/complications
Ovary/blood supply*
Postoperative period
Renal artery/injuries*
Tissue donors
Treatment outcome
Vascular grafting/methods*
Warm ischemia
